# Editorial: Proceedings of anatomy 2024 – tripartite meeting: the role of medical embryology for interdisciplinary research

**DOI:** 10.3389/fcell.2026.1929789

**Published:** 2026-08-26

**Authors:** Christian Schöfer, Gabriela Morosan-Puopolo, Wolfgang J. Weninger

**Affiliations:** 1 Division for Cell and Developmental Biology, Center for Anatomy and Cell Biology, Medical University of Vienna, Vienna, Austria; 2 Department of Anatomy and Molecular Embryology, Institute of Anatomy, Ruhr University Bochum, Bochum, Germany; 3 Division of Anatomy, Center for Anatomy and Cell Biology, Medical University of Vienna, Vienna, Austria

**Keywords:** embryo engineering, fertility research, histopathology, human assisted reproduction, imaging, molecular mechanism, organoids, regenerative medicine

The field of embryology encompasses a wide range of Research Topic and fields. Some areas of research focus on the elucidation of fundamental principles that govern developmental processes, others aim to understand the etiology of malformations, while still others integrate the insights gained by the former for use in the clinical setting. In this regard, the German-speaking Anatomy Societies, namely the Anatomische Gesellschaft (Germany), the Society Anatomy Austria, and the Swiss Society for Anatomy, Histology and Embryology, held their Tripartite Meeting at the Medical University of Graz, Austria from September 25 to 27, 2024. The diverse fields of medical embryology presented therein are reflected in this dedicated Research Topic of Frontiers of Cell and Developmental Biology. A total of nine articles from international author teams are presented, covering a range of Research Topic from basic research to the clinical implications of medical embryology.

In the domain of basic research, Schumann et al. investigated the orobasal organ, also known as the organ of Ackerknecht, in mice. The presence of this organ, which typically consists of variable epithelial invaginations behind the medial mandibular incisors, has been documented in many mammals, including humans and numerous rodents. However, only a single report is available for mice, wherein it was observed that the organ is absent in adult Japanese dance mice (Nishiyama, 1933). Schumann et al. studied the presence of the organ in *M. musculus,* and were able to identify the organ during embryonic development for the first time in mice. Further studies should reveal if the organ persists into adulthood in mice and to clarify developmental mechanisms and functional significance.

Maurer-Gesek et al. present a detailed study of the 3D-development of the inner ear of mice, from the formation of the otic vesicle onwards (E9.5-E15.5), using the method of high-resolution episcopic imaging (HREM). The authors made a detailed 3D-map of the membranous inner ear development, achieving an unprecedented level of detail. Furthermore, the authors analyzed mutant embryos with loss of function of genes relevant to inner ear development and were able to map distinct inner ear malformations in these animals. The presented 3D maps of the normal inner ear as well as of the malformations might help to understand the pathomechanism of the relatively high prevalence of human ear congenital malformations that can lead to congenital hearing loss (Morton and Nance, 2006).

In a further basic research study, Rockel et al. investigated the presence of macrophages in cardiac organoids. Cardiac organoids have received tremendous interest following their initial description (Hofbauer et al., 2021). To date, there has been no description of tissue-resident macrophages developing in cardiac organoids. However, they are present in normal heart tissue, where they were shown to play important roles in development, tissue homeostasis and improvement of contractile function. The authors employed an approach involving iPSC-derived cardiac organoids to demonstrate the differentiation of macrophages originating from hemogenic endothelium and their integration into cardiac muscle. Consequently, their physiologic model accurately replicates the intricate interactions within the heart, thereby facilitating a profound understanding of the role of macrophages in cardiac development and disease.

In addressing development at the molecular level, Vcelkova and Latos undertake a review of the rapidly expanding body of knowledge on the importance of transcription factor binding to *cis*-regulatory elements, including enhancers and silencers, for trophoblast lineage specification in human and rodent placentas. The precise spatiotemporal regulation of gene expression is required to give rise to cyto-, syncytio-, and extravillous trophoblast populations. The current objective of the field is to establish a comprehensive landscape of the gene regulatory network that acts in trophoblast lineage specification. The authors conclude that in future, systems-level analysis will be pivotal to achieving a detailed understanding of the regulatory networks controlling normal placental development and function, as well as aberrations resulting in a variety of disorders.

A concise mini-review on the structure of the human placenta is presented by Barapatre and Frank. This review emphasizes the value of 3D microscopy for a quantitative approach to placental villous analysis from a pathological perspective. The assessment of placental histopathology is typically conducted using qualitative guidelines that are defined in the Amsterdam criteria (Khong et al., 2016). Here, the authors explore the benefits of 3D microscopy in the analysis of various pathological placental conditions, including fetal growth restriction, preeclampsia, and gestational diabetes mellitus. Distinct yet partially overlapping microstructural pathologies can be identified and quantified, thereby underscoring the significance of quantitative 3D analysis for our understanding of human placental disease mechanisms.

Yang et al. investigated the correlation between isolated congenital hypospadias and the expression of the androgen receptor (AR). According to the findings of (Springer, van den Heijkant and Baumann, 2016), the prevalence of the condition in China is 3.9 cases per 10,000 births. Until now, the etiology of hypospadias has remained unknown but a correlation with the expression level of the androgen receptor (AR) has been reported. However, conflicting data were found as to whether AR levels are increased or decreased. The authors compared the expression levels of AR in the preputial epithelia of normal males (i.e. treated for phimosis) and patients with isolated hypospadias using immunohistochemistry. Levels of AR intensity were found to be significantly lower in cases of hypospadias than in the control group. This finding provides evidence in support of a negative correlation between levels of AR and the formation of hypospadias, thereby implicating AR deficiency in the pathogenesis of the condition.

In a retrospective study in the area of assisted reproduction, Fan et al. examined the ploidy levels of embryos of female thalassemia patients conceived by *in vitro* fertilization (IVF) at a fertility clinic in Guangzhou, southeast China. Thalassemia is an autosomal recessively inherited disease that is common in southeast China (Cai et al., 2025). The authors performed NGS sequencing of isolated blastomeres in order to test for chromosomal ploidy. As a result, the authors found no significant differences in embryonic ploidy between the offspring of women who were carriers and those who were not, with the exception of slightly diminished euploidy observed in embryos of mothers affected with thalassemia aged 35–40 years. The data suggest that thalassemia of the mother does not generally affect the chromosomal integrity of embryos. However, older females may require careful counselling.

Finally, two extensive reviews by the same workgroup highlight the transfer of basic science results to the clinical applicability. In the first paper, Soczyńska et al., provide a comprehensive overview on preimplantation diagnosis and potential applications of gene editing for fertility treatments starting with historical methodologies. The second review, by Soczyńska et al., focuses on the fields of medical embryology and regenerative medicine. It discusses the importance of interdisciplinary collaboration for the translation of basic research findings into the clinical setting. Particular emphasis is placed on the connection between embryology and tissue regeneration, integrating the growing knowledge gained from organoid and embryoid techniques, as well as stem cell treatment, to enhance tissue regeneration. Notwithstanding, in both papers the authors also discuss and review the ethical and legal aspects that are intrinsically interwoven with these techniques.
